# Supplementary material for: Rescue of ApoE4-related lysosomal autophagic failure in Alzheimer’s disease by targeted small molecules
Source: Commun Biol. 2024 Jan 8;7:60. doi: 10.1038/s42003-024-05767-9 (PMC10774381; doi:10.1038/s42003-024-05767-9)
Supplement: Supplementary file 2 — Supplementary information [file 42003_2024_5767_MOESM2_ESM.pdf]

SUPPLEMENTARY INFORMATION

Rescue of ApoE4-related lysosomal autophagic failure in Alzheimer's disease by targeted small molecules

Meenakshisundaram Balasubramaniam*[1], Jagadeesh Narasimhappagari[1], Ling Liu[1], Akshatha Ganne[1], Srinivas Ayyadevara [1, 2], Ramani Atluri[1], Haarika Ayyadevara[3], Guy Caldwell[4], Robert J Shmookler Reis[1, 2], Steven W Barger[1, 2], W. Sue T Griffin*[1, 2].

[1] *Department of Geriatrics, University of Arkansas for Medical Sciences, Little Rock, USA.*

[2] *Central Arkansas Veterans Healthcare System, Little Rock, USA.*

[3] *University of Arkansas, Fayetteville, Fayetteville, AR, 72701, USA.*

[4] *University of Alabama, Tuscaloosa, AL, 35487, USA.*

*Correspondence: mbalasubramaniam@uams.edu (M.B.), and GriffinSueT@uams.edu (W.S.T.G.)

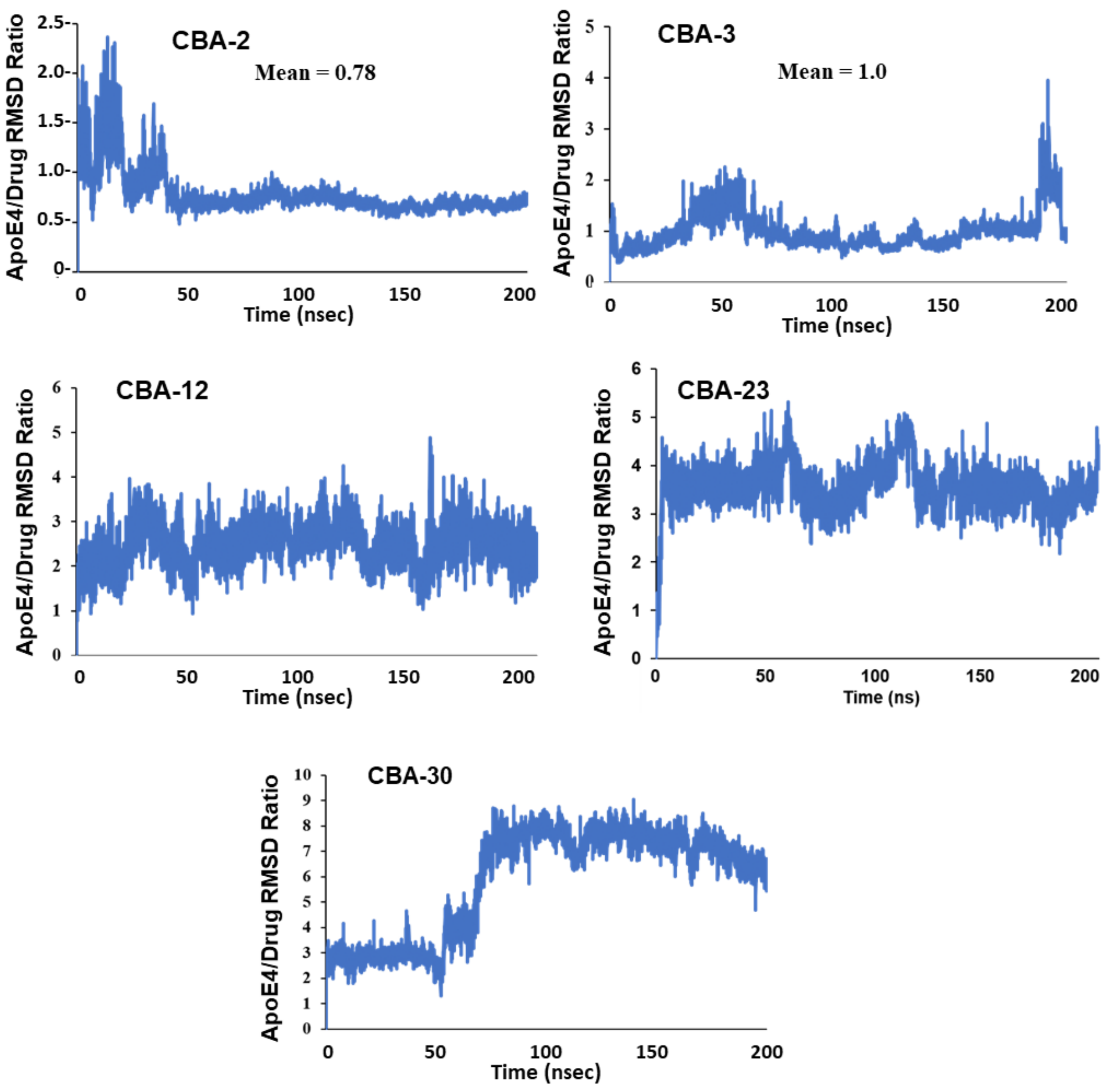

Supplementary Figure 1: Root Mean Square Deviation (i.e., protein to ligand RMSD) calculated from 200-ns MD simulation trajectories for the target ligands assessed.

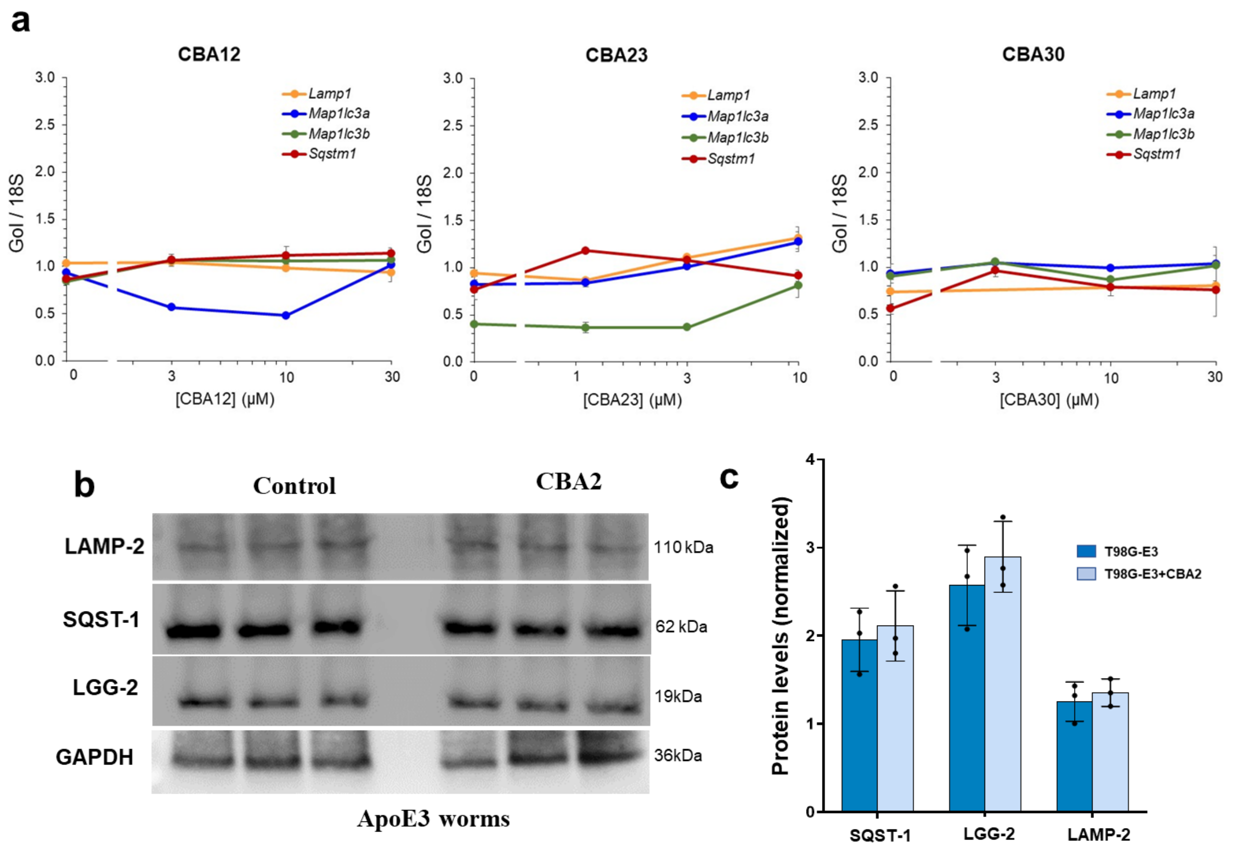

Supplementary Figure 2: (**a**) Primary astrocytes from ApoE-TR4 mice were exposed to low-glucose medium, and CBA12, -23, or -30 was applied at the indicated concentrations. After 20 h, mRNA levels of *Lamp1, Map1lc3a, Map1lc3b,* and *Sqstm1* were determined by qRT-PCR. (b) Western-blot analysis of key autophagy proteins in worms expressing either ApoE3 or ApoE4, ± CBA2. Exposure to CBA2 significantly increased levels of C.elegans SQST-1, LGG-2, and LAMP-2. (**c**) Histogram shows normalized band intensities calculated from western-blot images.

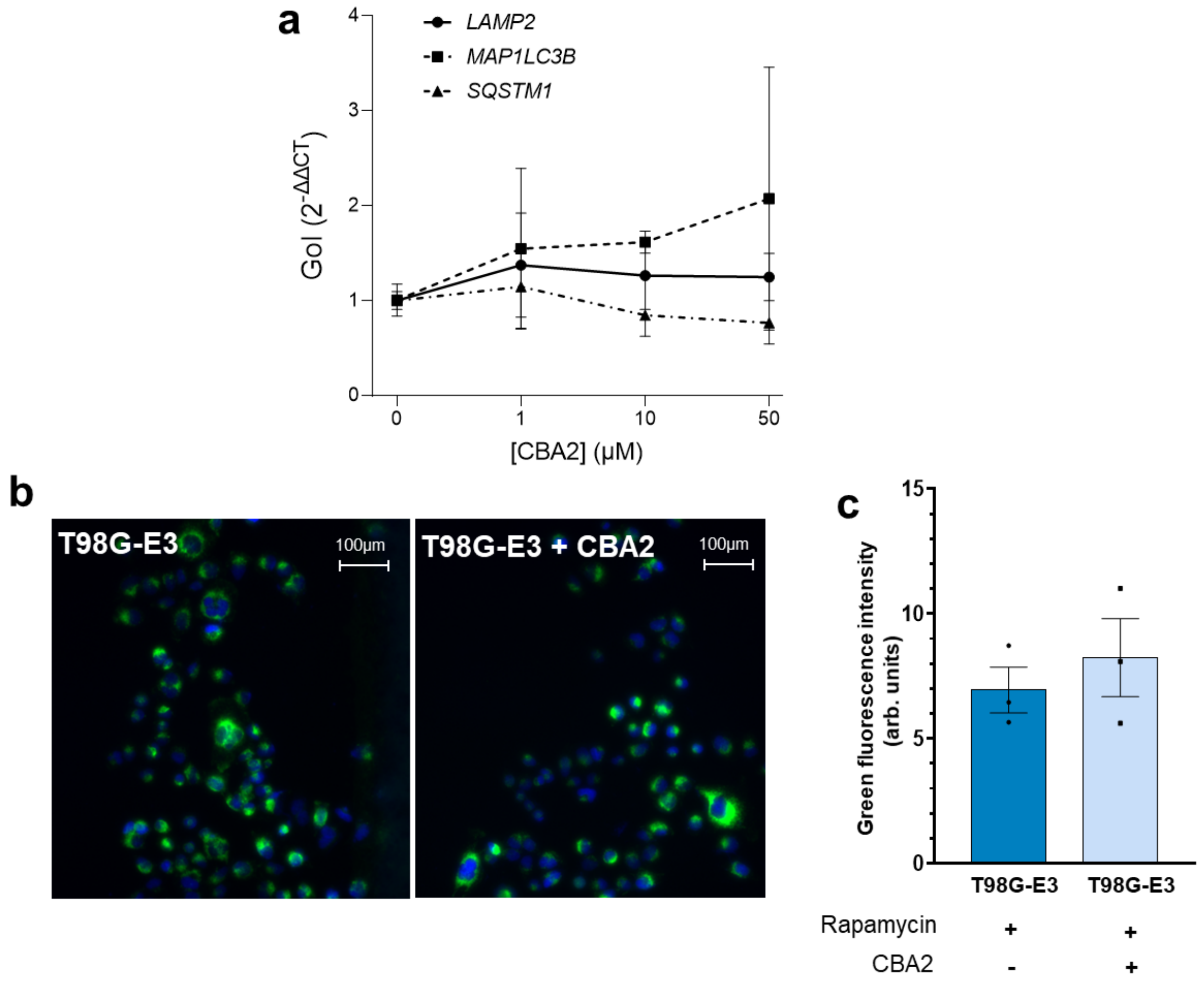

Supplementary Figure 3: (**a**)T98G-E3 cells were treated with CBA2 at the indicated concentrations. After 20 h, mRNA levels of *LAMP1, MAP1LC3A,* and *SQSTM1* were determined by qRT-PCR. (**b**) Images of T98G-E3 cells treated with rapamycin ± CBA2 treatment. (**c**) Histogram of mean normalized green intensity per cell calculated from fluorescent images, error bars represent ± SEM; *N*=3 repeats, represented as individual data points.

Dose (μmol/kg)	0	25	50	100
ALB (g/dL)	3.55	4	3.8	3.15
ALP (U/L)	71	79.5	68.5	54
ALT (U/L)	46	46	39	40
AMY (U/L)	760	816.5	928	719
TBIL (mg/dL)	0.2	0.25	0.25	0.3
BUN (mg/dL)	22	35	20	16
CA (mg/dL)	9.9	10.45	9.8	9.8
PHOS (mg/dL)	6.75	6.15	5.55	5.05
CRE (mg/dL)	0.35	0.25	0.25	0.35
GLU (mg/dL)	191	186.5	228.5	123
Na+ (mmol/L)	149	158.5	148.5	150
K+ (mmol/L)	4.65	5.3	4.6	5.3
TP (g/dL)	5.35	5.9	5.45	5.45
GLOB (g/dL)	1.8	1.85	1.6	2.25
HEM	0	0	1	0

Supplementary Table 1. Mouse toxicology results for CBA2

Supplementary Figure 4: Original uncropped western blot images

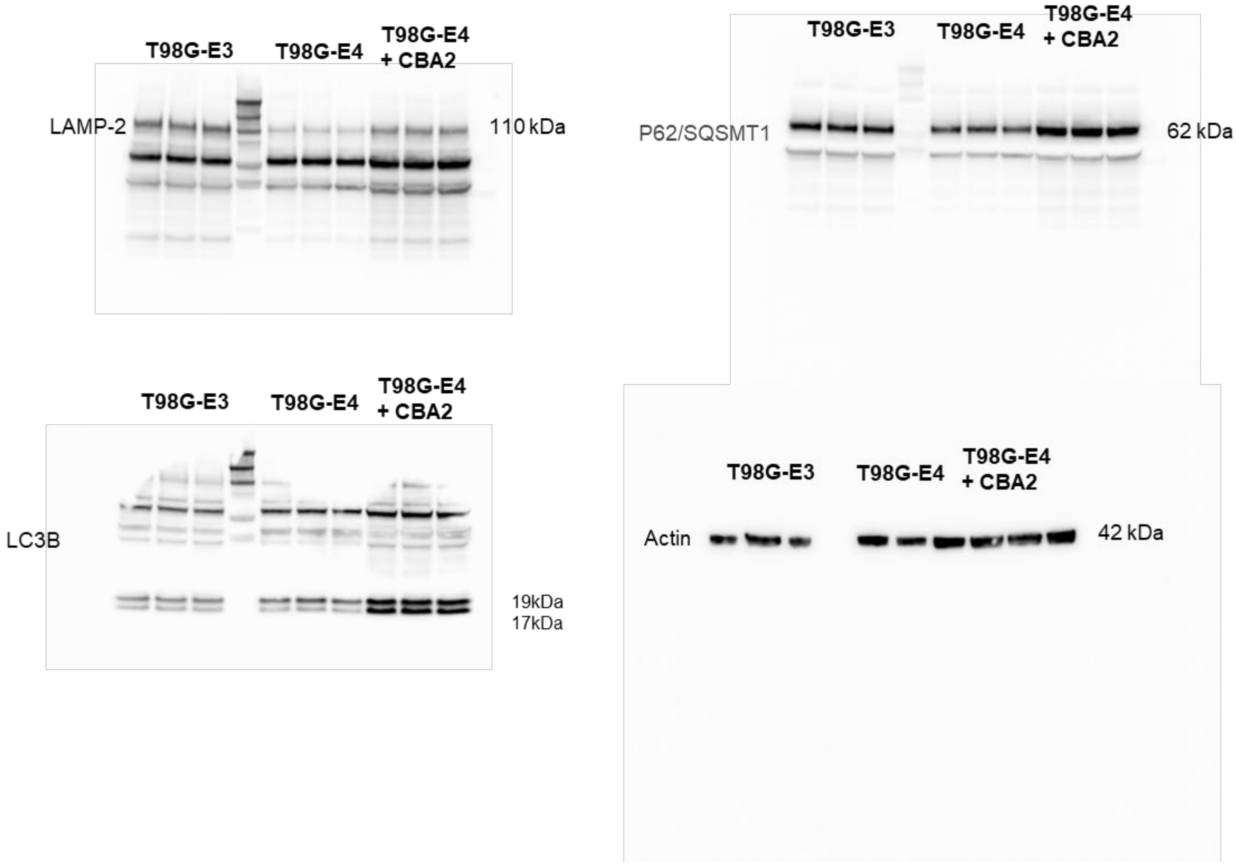

Figure 4e (original blots)

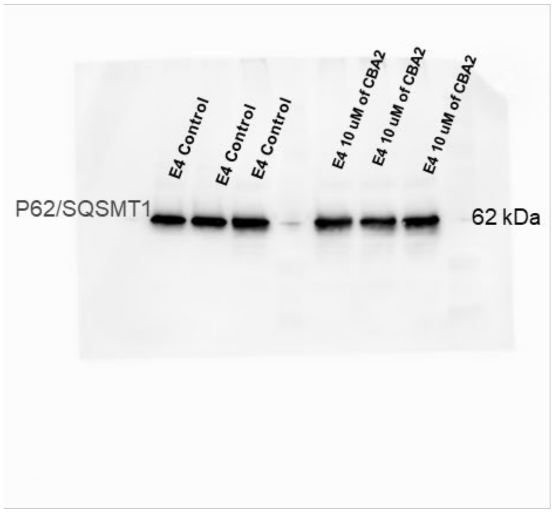

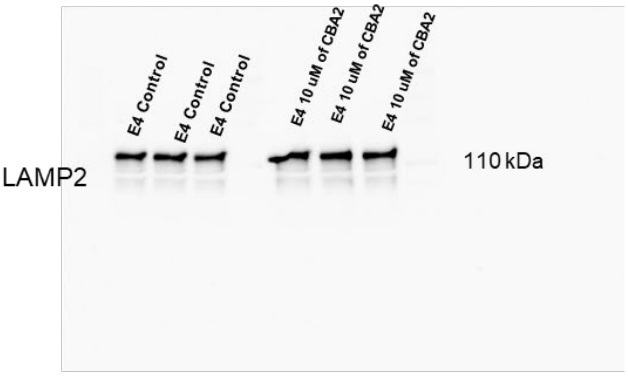

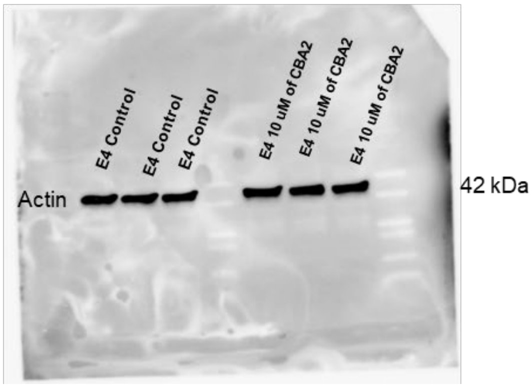

Figure 5d (original blots)
