# Supplementary material for: Rescue of ApoE4-related lysosomal autophagic failure in Alzheimer’s disease by targeted small molecules
Source: Commun Biol. 2024 Jan 8;7:60. doi: 10.1038/s42003-024-05767-9 (PMC10774381; doi:10.1038/s42003-024-05767-9)
Supplement: Supplementary file 3 — Reporting Summary [file 42003_2024_5767_MOESM3_ESM.pdf]

nature portfolio

Corresponding author(s): DBPR COMMSBIO-23-3068A

Last updated by author(s): 2023/11/06

Reporting Summary

Nature Portfolio wishes to improve the reproducibility of the work that we publish. This form provides structure for consistency and transparency in reporting. For further information on Nature Portfolio policies, see our Editorial Policies and the Editorial Policy Checklist.

Statistics

For all statistical analyses, confirm that the following items are present in the figure legend, table legend, main text, or Methods section.

n/a	Confirmed	
☐	☒	The exact sample size (*n*) for each experimental group/condition, given as a discrete number and unit of measurement
☐	☒	A statement on whether measurements were taken from distinct samples or whether the same sample was measured repeatedly
☐	☒	The statistical test(s) used AND whether they are one- or two-sided<br>*Only common tests should be described solely by name; describe more complex techniques in the Methods section.*
☐	☒	A description of all covariates tested
☐	☒	A description of any assumptions or corrections, such as tests of normality and adjustment for multiple comparisons
☐	☒	A full description of the statistical parameters including central tendency (e.g. means) or other basic estimates (e.g. regression coefficient) AND variation (e.g. standard deviation) or associated estimates of uncertainty (e.g. confidence intervals)
☒	☐	For null hypothesis testing, the test statistic (e.g. *F*, *t*, *r*) with confidence intervals, effect sizes, degrees of freedom and *P* value noted<br>*Give P values as exact values whenever suitable.*
☒	☐	For Bayesian analysis, information on the choice of priors and Markov chain Monte Carlo settings
☒	☐	For hierarchical and complex designs, identification of the appropriate level for tests and full reporting of outcomes
☒	☐	Estimates of effect sizes (e.g. Cohen's *d*, Pearson's *r*), indicating how they were calculated

Our web collection on statistics for biologists contains articles on many of the points above.

Software and code

Policy information about availability of computer code

Data collection	High-throughput virtual screening of small molecules were conducted using AutoDock-Vina (open-source) package. Molecular dynamic models were generated using GROMACS (open-source) simulation package.
Data analysis	All data analysis were obtained from either Microsoft Excel, or from GraphPad prism

For manuscripts utilizing custom algorithms or software that are central to the research but not yet described in published literature, software must be made available to editors and reviewers. We strongly encourage code deposition in a community repository (e.g. GitHub). See the Nature Portfolio guidelines for submitting code & software for further information.

Data

Policy information about availability of data

All manuscripts must include a data availability statement. This statement should provide the following information, where applicable:

- Accession codes, unique identifiers, or web links for publicly available datasets
- A description of any restrictions on data availability
- For clinical datasets or third party data, please ensure that the statement adheres to our policy

Initial coordinates, simulation input files, and coordinate files associated with in silico analyses are available from the corresponding author upon request. No other data associated with this study comprise large sets that would be valuable for data mining.

Research involving human participants, their data, or biological material

Policy information about studies with human participants or human data. See also policy information about sex, gender (identity/presentation), and sexual orientation and race, ethnicity and racism.

Reporting on sex and gender	Not applicable
Reporting on race, ethnicity, or other socially relevant groupings	Not applicable
Population characteristics	Not applicable
Recruitment	Not applicable
Ethics oversight	Not applicable

Note that full information on the approval of the study protocol must also be provided in the manuscript.

Field-specific reporting

Please select the one below that is the best fit for your research. If you are not sure, read the appropriate sections before making your selection.

☒ Life sciences ☐ Behavioural & social sciences ☐ Ecological, evolutionary & environmental sciences

For a reference copy of the document with all sections, see nature.com/documents/nr-reporting-summary-flat.pdf

Life sciences study design

All studies must disclose on these points even when the disclosure is negative.

Sample size	There was little or no preliminary data on which to base a power analysis. After conducting experiments, a post-hoc power analysis was performed to determine validity of the results.
Data exclusions	No data were excluded.
Replication	Each type of experiment was performed >2 times except for the in-vivo study on mice. IACUC opinion considered a repeat of the experiment incongruous with the mandate to test the hypothesis with the smallest possible impact on animal welfare.
Randomization	For the in-vivo study, mice were selected for treatment groups via a random-number generator. Other experiments with cell cultures are inherently consistent, obviating a regimented randomization protocol.
Blinding	Technicians evaluating chemotaxis in C. elegans were unaware of the genotype or treatment. Other endpoints were considered sufficiently objective as to make blinding unnecessary.

Reporting for specific materials, systems and methods

We require information from authors about some types of materials, experimental systems and methods used in many studies. Here, indicate whether each material, system or method listed is relevant to your study. If you are not sure if a list item applies to your research, read the appropriate section before selecting a response.

Materials & experimental systems

n/a	Involved in the study	
☐	☒	Antibodies
☐	☒	Eukaryotic cell lines
☒	☐	Palaeontology and archaeology
☐	☒	Animals and other organisms
☒	☐	Clinical data
☒	☐	Dual use research of concern
☒	☐	Plants

Methods

n/a	Involved in the study	
☒	☐	ChIP-seq
☒	☐	Flow cytometry
☒	☐	MRI-based neuroimaging

Antibodies

Antibodies used	Anti-SQSTM1 / p62 antibody, mouse monoclonal from Abcam Catolog-(ab56416); LAMP-2/CD107b Antibody, rabbit monoclonal from

Antibodies used	Novus Biologicals Catolog-NBS 67298; Anti-LC3B antibody, rabbit Polyclonal Antibody from Abcam, Catalog-ab63817; GAPDH mouse monoclonal antibody from Santacruz, catalog number-SC-47724; and Beta Actin rabbit monoclonal from cell signaling Technologies, Catalog No. 8457S
Validation	Care was taken to confirm that the detection signal was at the correct relative mobility on SDS-PAGE. We executed no additional validation of the antibodies as they are commercially available and have been validated in multiple publications.

Eukaryotic cell lines

Policy information about cell lines and Sex and Gender in Research

Cell line source(s)	T98G parental cells were obtained from American Type Culture Collection. Stable transfectants overexpressing either ApoE3 or ApoE4 were generated in our laboratory.
Authentication	Authentication of the T98G cells was provided by ATCC.
Mycoplasma contamination	The cell lines are free from Mycoplasma contamination.
Commonly misidentified lines (See ICLAC register)	T98G cells were not found in the ICLAC Registry version 12.

Animals and other research organisms

Policy information about studies involving animals; ARRIVE guidelines recommended for reporting animal research, and Sex and Gender in Research

Laboratory animals	Targeted Replacement (TR) mice expressing either human APOE3 or APOE4 were obtained from Taconic. C.elegans strains were kindly provided by Dr. Guy Caldwell from University of Alabama, USA
Wild animals	Not applicable
Reporting on sex	A total of 23 mice were used for the experiments in this study, all of which were male. The purpose of this experiment was to establish an effect in a model that might be used for future studies testing interactions between ApoE and the accumulation of amyloid beta-peptide, to model one aspect of Alzheimer pathogenesis. In our preferred model of amyloid accumulation, females accumulating amyloid do not exhibit a behavioral or physiological phenotype. Therefore, we have initiated our pilot studies in males for the sake of relevance to future studies.
Field-collected samples	N.A.
Ethics oversight	The experiments were reviewed and approved by the Animal Care and Use Committee of the Central Arkansas Veterans Healthcare System, a local unit of the U.S. Department of Veterans Affairs.

Note that full information on the approval of the study protocol must also be provided in the manuscript.

Plants

Seed stocks	N.A.
Novel plant genotypes	N.A.
Authentication	N.A.
